# Dietary Protection Against Free Radicals: A Case for Multiple Testing to Establish Structure-activity Relationships for Antioxidant Potential of Anthocyanic Plant Species

**DOI:** 10.3390/ijms10031081

**Published:** 2009-03-11

**Authors:** Martin Philpott, Chiara Cheng Lim, Lynnette R. Ferguson

**Affiliations:** Discipline of Nutrition, Faculty of Medical & Health Science, The University of Auckland, Private Bag 92019, Auckland, New Zealand; E-Mails: m.philpott@auckland.ac.nz (M.P.); chiara.lim@hotmail.com (C.L.)

**Keywords:** Anthocyanin, reactive oxygen species, antioxidant, free radical scavenging, single cell gel electrophoresis assay

## Abstract

DNA damage by reactive species is associated with susceptibility to chronic human degenerative disorders. Anthocyanins are naturally occurring antioxidants, that may prevent or reverse such damage. There is considerable interest in anthocyanic food plants as good dietary sources, with the potential for reducing susceptibility to chronic disease. While structure-activity relationships have provided guidelines on molecular structure in relation to free hydroxyl-radical scavenging, this may not cover the situation in food plants where the anthocyanins are part of a complex mixture, and may be part of complex structures, including anthocyanic vacuolar inclusions (AVIs). Additionally, new analytical methods have revealed new structures in previously-studied materials. We have compared the antioxidant activities of extracts from six anthocyanin-rich edible plants (red cabbage, red lettuce, blueberries, pansies, purple sweetpotato skin, purple sweetpotato flesh and Maori potato flesh) using three chemical assays (DPPH, TRAP and ORAC), and the *in vitro* Comet assay. Extracts from the flowering plant, lisianthus, were used for comparison. The extracts showed differential effects in the chemical assays, suggesting that closely related structures have different affinities to scavenge different reactive species. Integration of anthocyanins to an AVI led to more sustained radical scavenging activity as compared with the free anthocyanin. All but the red lettuce extract could reduce endogenous DNA damage in HT-29 colon cancer cells. However, while extracts from purple sweetpotato skin and flesh, Maori potato and pansies, protected cells against subsequent challenge by hydrogen peroxide at 0°C, red cabbage extracts were pro-oxidant, while other extracts had no effect. When the peroxide challenge was at 37°C, all of the extracts appeared pro-oxidant. Maori potato extract, consistently the weakest antioxidant in all the chemical assays, was more effective in the Comet assays. These results highlight the dangers of generalising to potential health benefits, based solely on identification of high anthocyanic content in plants, results of a single antioxidant assay and traditional approaches to structure activity relationships. Subsequent studies might usefully consider complex mixtures and a battery of assays.

## 1. Introduction

Essential and life sustaining biological processes such as cellular metabolism cannot occur without the formation of reactive oxygen species (ROS). However, the uncontrolled increase of ROS is strongly associated with the etiology and pathophysiology of a number of chronic human diseases such as inflammation, viral infections, cancers, autoimmune, neurodegenerative, cardiovascular and digestive system disorders, as well as premature aging. High levels of ROS have been reported to alter the structure and function of cell membranes, adversely affecting cell function [1]. The human diet is the primary source of antioxidants that protect against exposure to peroxidized compounds in the digestive tract. Diets rich in antioxidants due to high consumption of fruits, vegetables cereals and wines have been linked to reduced incidence of these chronic degenerative diseases [2].

**Figure 1.**
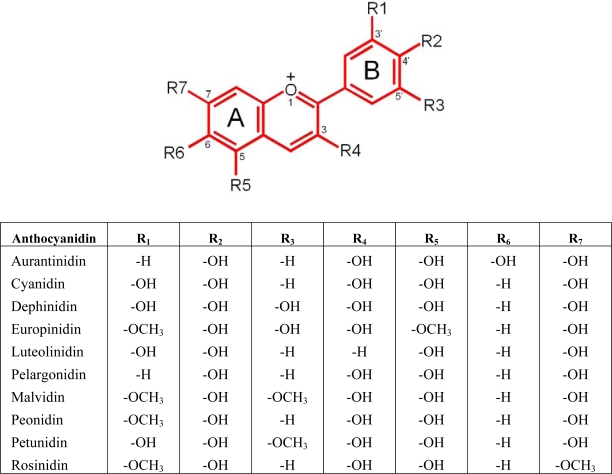
The basic structure of an anthocyanin.

In addition to their aesthetic qualities in food, various examples of major plant pigments, including polyphenols such as anthocyanins, have been shown to have metabolic and nutritional benefit, partly through their antioxidant effects [3]. Anthocyanins are glycosides of polyhydroxyl and polymethoxyl derivatives of flavylium salts, that are responsible for the intense purple, blue or red pigmentation found in various plants (Figure 1) [4].

Circulatory and cardiovascular diseases may be reduced by the antioxidant actions of anthocyanins, as demonstrated by the increased resistance to hydrogen peroxide-induced ROS generation of red blood cells (RBC) treated with these compounds. Many anthocyanins appear to show superior antioxidant properties to other related polyphenols [5] as well as to well characterised antioxidants such as ascorbic acid or α-tocopherol [6,7]. Radical scavenging activity is usually increased in proportion to a decrease in glycosylation and an increase in the number of hydroxyl groups. Specifically, dihydroxy substitutions in the B ring and fusion of the A and B rings appeared to result in an increase in antioxidant capacity as measured in a single assay [8]. However, it is important to recognise that there are different types of antioxidants, and such a chemical analysis neglects activities on cellular enzymes [9]. Given that these latter effects are likely to be long-lived, they may be considerably more important than free radical scavenging for sustainable human health.

Anthocyanic vacuolar inclusions (AVIs) contain high numbers of anthocyanin molecules, stacked onto a protein matrix [10]. They are contained within plant cell vacuoles but are not membrane bound despite a distinct round outline when viewed under the microscope [11]. It is hypothesized that AVIs could act as superantioxidants due to the sheer concentration of anthocyanins in a relatively small area. On the other hand the lack of surface area could hinder the potential antioxidant capacity of AVIs and, for this reason, free anthocyanins would be the better antioxidant.

If an anthocyanin-rich food is to have significant influence on health, it becomes important to consider levels in commonly eaten foods that could realistically be incorporated into the human diet. Some good examples are summarised in Table 1.

Lettuce and cabbage are common dietary items for which red variants have good anthocyanin levels, and are commonly available in the food supply [12]. Blueberries have been extensively characterised as excellent antioxidant sources[13]. Although not a dietary mainstay, deep purple pansies are good anthocyanin sources that are edible. We have previously highlighted some intensely purple coloured sweetpotato as excellent anthocyanin sources, for which both skin and flesh are edible [14,15]. Taewa is a collective noun referring to the ‘Maori’ potato; a collection of varieties of Solanum tuberosum now cultivated by Maori for at least 200 years. The variety Tutaekuri (Urenika) describes a long yam-like tuber with dark purple skin and flesh, whose intense colouration relates to high anthocyanin levels [16,17].

An additional difficulty in determining human health implications of anthocyanin-rich foods, is that the anthocyanins are not found as isolated compounds, but rather as a component of often complex mixtures. Possible pro-oxidant actions at certain concentrations also need to be considered [18]. In this study, we have compared the antioxidant properties of anthocyanin-rich extracts (AREs) from five good dietary sources, with those of the flowers of *Viola × wittrockiana* (pansy; an edible food plant) and *Eustoma grandiflorum* (lisianthus), the latter of which is known to have anthocyanins in an AVI [10]. Three different chemical assays were used to complement a cellular assay that tested their ability to protect against DNA damage induced by hydrogen peroxide. The Total Reactive Antioxidant Potential (TRAP) assay was also used to compare the antioxidant properties of intact AVIs to that of liberated anthocyanins. Purple lisianthus petals were used as a convenient source of AVIs, as these give large yields. Although several of the plants tested may have superficially appeared as comparable dietary sources of antioxidants, significant differences became apparent from the various assay methods. We could not have predicted the results based on prior knowledge of anthocyanin composition alone.

## 2. Results and Discussion

### 2.1. Anthocyanin levels

**Figure 2.**
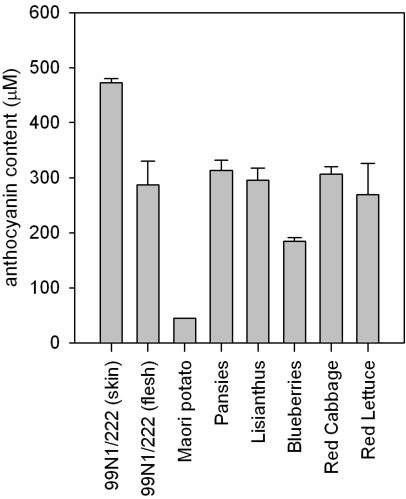
Anthocyanin content of the indicated sources. Anthocyanins from 1 g of fresh material was extracted into 10 mL of acidified methanol. Vertical lines represent the SEM.

Anthocyanin contents of 1 g of fresh material as extracted into 10 mL of acidified methanol are compared in Figure 2.

This figure illustrates that 99N1/222 flesh, pansies, lisianthus, red cabbage and red lettuce had similar levels of anthocyanins. However, as compared with extracts from these five plants, the extract from Maori potato flesh had an approximately 6-fold lower anthocyanin content, that from the blueberry extracts showed approximately two thirds of the anthocyanin content, while the 99N1/222 skin extract had an anthocyanin content more than 50% higher.

### 2.2. DPPH Assay

**Figure 3.**
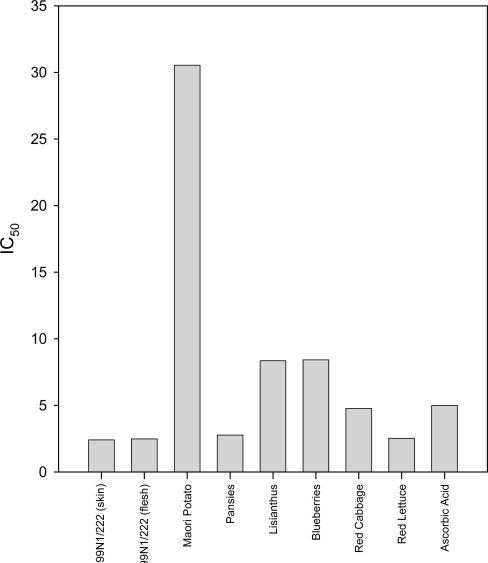
Bar graph showing IC_50_ values for each extract from the DPPH assay. The IC_50_ values represent the volume of extract required to reduce the absorbance of the DPPH radical by half and were calculated from the average of duplicate absorbances.

Antioxidant values in the DPPH assay were calculated as the volume of extract required to scavenge half the DPPH radical (IC_50_) and are displayed in Figure 3. In this figure, smaller values represent superior antioxidants. Most of the extracts had similar IC_50_ values, except for the Maori potato, which had about a 13-fold higher IC_50_ value compared to 99N1/222 skin extract, which had the lowest IC_50_ (Figure 3). Five of the eight extracts, namely the 99N1/222 skin, flesh, pansies, red cabbage and red lettuce extracts, were better antioxidants than the 1 mM ascorbic acid standard, while the lisianthus and blueberry extracts showed less antioxidant activity than the standard.

### 2.3. TRAP Assay

**Figure 4.**
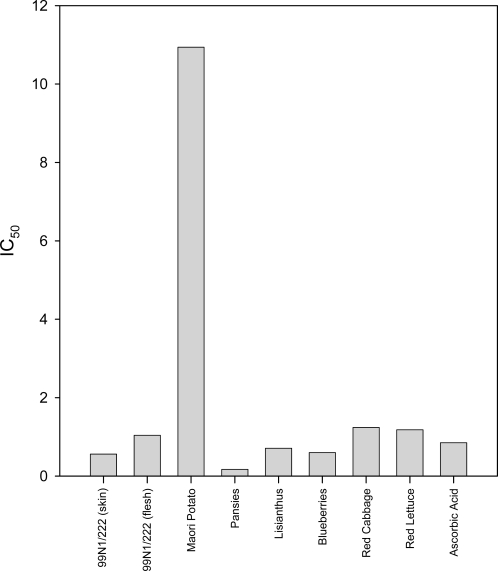
Bar graph showing IC_50_ values for each anthocyanin extract from the TRAP assay. The IC_50_ values represent the volume of extract required to reduce the absorbance of the TRAP reaction mixture by half, and were calculated from the average of duplicate absorbances.

Like the DPPH assay, the TRAP assay determines the IC_50_ of the extracts (Figure 4). The TRAP assay again showed that most extracts had similar IC_50_ values, except those from the Maori potato, which had about a sevenfold higher IC_50_ value in comparison with pansy extract which had the lowest IC_50_ Four of the eight extracts, namely the 99N1/222 skin, pansies, lisianthus, and blueberry extracts were better antioxidants than the ascorbic acid standard, while the 99N1/222 flesh, red cabbage and red lettuce showed slightly less antioxidant activity than the standard.

This same assay was used to compare antioxidant activities of extracts and AVIs from the throat of the deep purple lisianthus, and from a cream lisianthus, after either 15 minutes or overnight (24 h) incubation at room temperature (Figure 4).

After 15 minutes, the TRAP activity of the free anthocyanic solution was higher as compared with extracted AVIs, and no activity was seen in the extract from the non-pigmented lisianthus. Radical scavenging by the free anthocyanins was saturated after 15 minutes, with no further increase after 24 hours. In contrast, intact AVIs continued to scavenge the ABTS radical, showing markedly superior total antioxidant activity compared to free anthocyanins after 24 hours. Unfortunately, seasonal differences in the varieties of lisianthus available have made it impossible to extract sufficient quantities of AVIs to repeat this observation.

### 2.4. ORAC Assay

**Figure 6.**
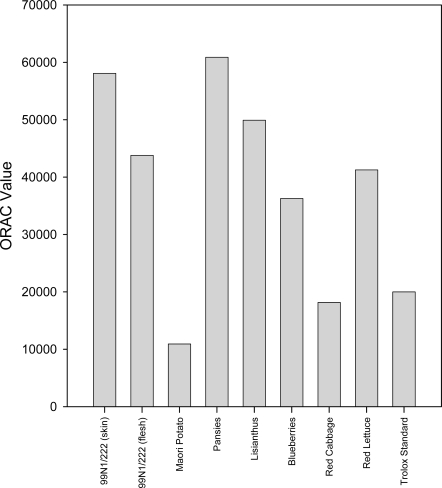
Bar graph showing ORAC values for each extract from the ORAC assay. The ORAC values represent the efficiency of each extract to inhibit peroxyl radical oxidation, thus R-PE fluorescence, and were calculated from the average of duplicate S values derived from the measurement of fluorescence.

Unlike the previous assays, the ORAC assay measures the area under the curve of the time course of extinction of the luminescent compound R-PE. Higher values, therefore, indicate better antioxidant activity. The ORAC assay reveals more variation in the antioxidant activities of each extract (Figure 6).

Six of the eight extracts, namely those from the 99N1/222 skin, flesh, pansies, lisianthus, blueberries and red lettuce extracts showed a higher ORAC value than the 1 mM Trolox^®^ standard (which has comparable antioxidant activity to 1 mM ascorbic acid). Red cabbage extract showed slightly lower antioxidant activity than the standard, while Maori potato extract was again the least effective antioxidant, about 6-fold lower than the pansy extract, which had the highest ORAC value.

### 2.5. Comet Assay

Three variations of the Comet assay were performed. In the first, cells were incubated for three days with or without the previously identified maximum non-toxic dose of the anthocyanic plant extracts. The cells were then assayed for DNA damage without further treatment (untreated group). In the other two groups, after three days of incubation with or without the extracts, the cells were resuspended in PBS and challenged with hydrogen peroxide for 15 minutes at either 0 ºC or 37 ºC (referred to as the 0 ºC treatment group and the 37 ºC treatment group, respectively). For each of the treatment groups, the average tail extent moments of 100 comets for the control cells (no extract) plus the cells incubated with the various extracts were calculated and are described below.

#### 2.5.1. Untreated Group

**Figure 7.**
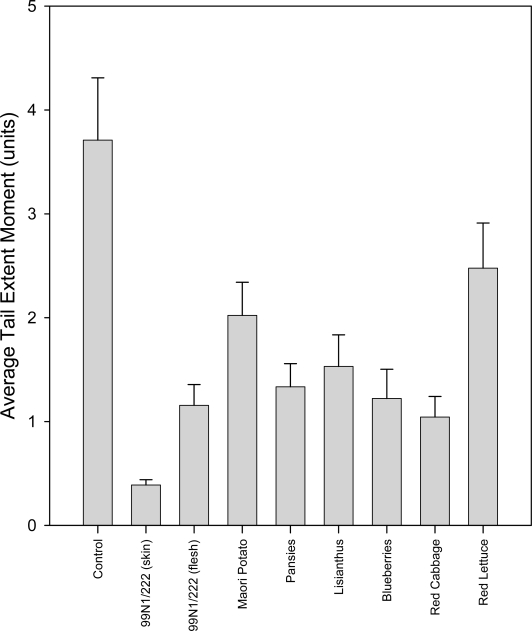
Bar graph showing the average tail extent moments and standard errors from 100 comets incubated in different anthocyanic plant extracts in the untreated group.

In the group that did not receive hydrogen peroxide treatment, all the extracts cause a reduction in average tail extent moment or tail length relative to the control (Figure 7). The tail moments show a variable degree of reduction, with the 99N1/222 skin showing the greatest decrease and red lettuce showing the smallest reduction. The average tail lengths present the same, but less pronounced, pattern and the frequency distribution patterns show that the data are normally distributed. DNA damage in the untreated control cells was low, but measurable (Table 2).

The majority of the extracts were able to provide a significant reduction (p≤0.05) in constitutive DNA damage, with only the red lettuce (p=0.098) extract unable to do so. The 99N1/222 skin, which had the best overall protection, was also significantly better than all the other extracts in terms of reducing background DNA damage (p≤0.01). 99N1/222 flesh and red cabbage extract was significantly better than the bottom ranked two extracts, Maori potato (p=0.023 and p=0.010, respectively) and red lettuce (p=0.006 and p=0.003, respectively), while pansy and blueberry extracts were significantly better than red lettuce (p=0.020 and p=0.016, respectively). The remaining extracts, from lisianthus, Maori potato and red lettuce, showed no significant differences in ability to protect against endogenous DNA damage (p>0.05).

#### 2.5.2. 0 °C Hydrogen Peroxide Treatment Group

In addition to the protection from endogenous free radicals examined in the previous section, the effects of the various extracts on subsequent challenge by exogenous hydrogen peroxide was also studied. By performing these assays at 0 ºC, enzymatic effects could be eliminated. Therefore, any antioxidant effects observed in response to the extracts would be due to direct scavenging of hydrogen peroxide entering the cells, or by prior lowering of the cell redox state so that it can absorb more ROS before damage occurred.

**Figure 8.**
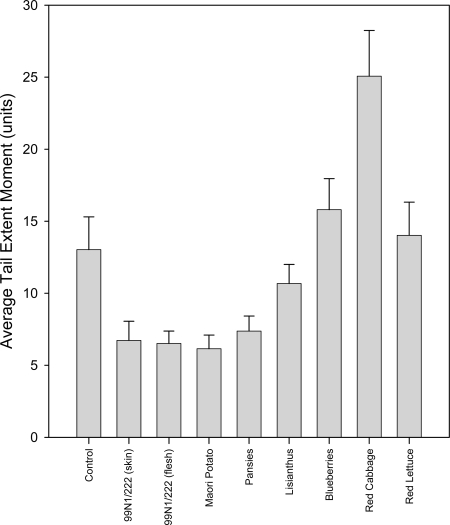
Bar graph showing the average tail extent moments and standard errors from 100 comets incubated in different anthocyanic plant extracts in the 20 μL H_2_O_2_ at 0 °C treatment group.

In the group that received hydrogen peroxide treatment at 0 ºC, the control had increased DNA damage over the controls in the non-peroxide treated group. However, not all the extracts were able to cause a reduction in average tail extent moment relative to the control (Figure 8).

Relative to the untreated cells, hydrogen peroxide increased DNA damage in the controls (p<0.0001) (Table 3).

Four of the extracts, 99N1/222 skin and flesh, Maori potato and pansies, significantly reduced hydrogen peroxide damage by approximately half (p<0.05). Lisianthus, red lettuce and blueberry extracts had no significant effect (p>0.05) and red cabbage extract actually caused an increase in DNA damage to about twice that of the controls (p=0.002).

#### 2.5.3. 37 ºC Hydrogen Peroxide Treatment Group

**Figure 9.**
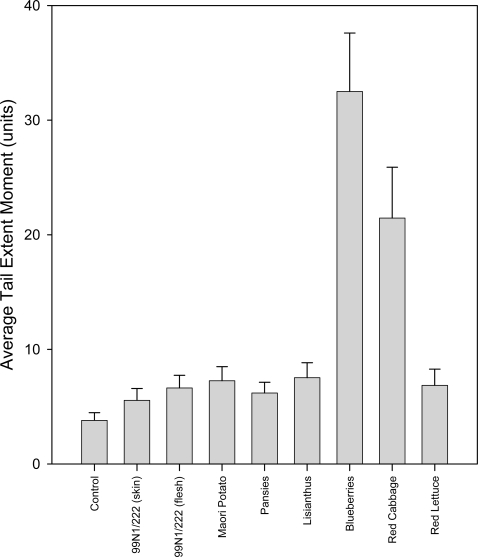
Bar graph showing the average tail extent moments and standard errors from 100 comets incubated in different anthocyanic plant extracts in the 20 μL H_2_O_2_ at 37 °C treatment group.

In the group that received hydrogen peroxide treatment at 37 ºC, the control cells showed the least amount of DNA damage (Figure 9).

Unexpectedly, none of the extracts were able to reduce DNA damage induced by hydrogen peroxide at 37 ºC to below the control (Table 4).

With the exception of 99N1/222 skin extract (p=0.159), all the extracts produced significantly more DNA damage relative to the control (p<0.05). Pansy, 99N1/222 flesh, Maori potato and lisianthus all produced DNA damage at 37 ºC that was indistinguishable from their respective values at 0 ºC (p>0.05). The red lettuce extract, while still inducing more DNA damage relative to the control (p=0.002), had less damage at 37 ºC than at 0 ºC (p=0.010). DNA damage at 37 ºC and 0 ºC with the red cabbage extract did not significantly alter (p=0.508). The blueberry extract, which showed neither protective nor deleterious effects relative to the control at 0 ºC (p=0.367), exhibited twice the DNA damage at 37 ºC than at 0 ºC (p=0.003).

#### 2.5.4. Comparison of 0 ºC and 37 ºC Hydrogen Peroxide Treatment Groups

The degree of hydrogen peroxide stimulated DNA damage in the presence of each of the extracts at both 0 ºC and 37 ºC is compared in Table 5.

The p-values show that DNA damage in the controls and red lettuce has significantly reduced at 37 ºC, but that damage induced by 99N1/222 skin and flesh, Maori potato, pansy, lisianthus and red cabbage extracts have stayed the same at both temperatures. In contrast, the damage induced by blueberries has significantly increased at 37 ºC in comparison to the 0 ºC treatment.

### 2.6. Discussion

The three chemical assays evaluated measure primary antioxidant activity of the anthocyanic extracts, while the Comet assay can also measure secondary effects. However, the chemical assays are quick and simple methods of measuring potential antioxidant activity, whereas utilising oxidation of cellular components can be time consuming to perform and not practical where large numbers of samples are involved

The common feature of the three chemical assays is their direct measurement of free radical scavenging efficiency of the extracts. The DPPH assay is the quickest and easiest assay to perform, but it diverges from biological conditions the most, using an artificial DPPH radical and methanol as the solvent [18]. This method is only able to measure direct reactions with the DPPH radical, which is dependent on the structure of an antioxidant compound and can only give a general indication of the radical scavenging abilities of antioxidants. However, it is a rapid and convenient method for screening many samples as well as not requiring expensive reagents or sophisticated equipment [22,23]. The TRAP (Total Reactive Antioxidant Potential) assay is also relatively quick and easy to carry out, with the advantage over the DPPH assay of being in aqueous conditions. However, the TRAP assay still utilizes a non-biological ABTS cation radical [24]. The TRAP and ORAC (Oxygen Radical Absorbance Capacity) are similar assays because they make use of the hydrogen atom transfer (HAT) reaction between an oxidant and a free radical. Both assays use AAPH [2,2′-azobis(2-amidino-propane) dihydrochloride] as a peroxyl radical generator, which is a commonly found free radical in the body [25]. However, in the TRAP assay, the peroxyl radical does not directly interact with the antioxidant extract. In a pre-incubation step, before the addition of the antioxidant species, AAPH-generated peroxyl radicals oxidise ABTS [2,2′-azinobis(3-ethylbenzothiazoline-6-sulfonate)] to generate the ABTS radical. The ability of an antioxidant to scavenge the pre-formed ABTS radical and the subsequent loss in absorbance at 734 nm, which is proportional to the antioxidant capacity of the antioxidant being tested[24], forms the basis of the TRAP assay. As with the DPPH assay, the TRAP assay is a quick and easy method, convenient when high sample numbers are being tested, but uses a non-biological radical for measuring antioxidant activity.

The ORAC assay measures the degree and length of time the extracts take to inhibit the action of an oxidizing agent. It therefore takes into account the kinetics of the reaction, unlike the other two assays, as well as being performed at a physiological pH and producing a biologically relevant radical, the peroxyl radical [26]. Since anthocyanin stability and therefore its antioxidant activity is sensitive to changes in temperature and pH, inappropriate conditions can greatly influence the result. The assay utilizes the fluorescent protein R-PE (R-phycoerythrin) as a detector of antioxidant activity. The peroxyl radicals generated by AAPH can either react with the antioxidant extract by removing a hydrogen atom from it or by damaging R-PE, resulting in a loss of fluorescence. The efficiency of the extract to inhibit the decline of R-PE fluorescence is measured [26]. In contrast to the DPPH and TRAP assays, the ORAC assay measures the antioxidant activity of the extracts against the biologically relevant peroxyl radical, as well as taking into account the kinetics of the chain-breaking reactions [27]. However, the ORAC assay does not measure the total antioxidant activity because other biologically relevant ROS exist, such as superoxide, the hydroxyl radical and singlet oxygen. Because different ROS have different reaction mechanisms, to completely determine antioxidant activity against a wide range of ROS, a more comprehensive set of assays need to be carried out [6].

Far more biologically relevant is the Comet assay, which visualizes DNA damage in single cells arising from the exposure to various combinations of antioxidants and ROS [28,29].. Cells are embedded in agarose coated slides and subjected to electrolysis, which causes the negatively charged DNA to migrate towards the anode. Damaged and fragmented DNA is able to migrate through the agarose faster, and can be visualized using ethidium bromide which intercalates within the DNA. An alkaline Comet assay was performed as opposed to the neutral Comet assay because this assay is able to detect single strand damage, due to denaturation of DNA, and therefore is more representative of actual DNA damage. Tail intensity represents the amount of DNA and, therefore, the degree of DNA damage, while the tail length gives an indication of fragment size since smaller fragments migrate faster and farther through the agarose [21]. Combined, these parameters give the tail extent moment which takes into account both the extent of DNA damage and fragment size [30].

The initial set of COMET assay results measure constitutive DNA damage in cells without exposure to an exogenous source of free radicals. They demonstrated that most of the extracts could protect the DNA from damage by endogenous free radicals generated by the cells during their 3 day incubation period. 99N1/222 skin offered the best protection of all of the extracts tested, while the red lettuce extract did not offer any significant protection relative to the controls. The remaining extracts, while showing significant improvement from the control, were in the middle ground in terms of their antioxidant capacities when comparing between extracts.

Of the extracts that were able to offer some protection against hydrogen peroxide challenge, none were able to completely protect, indicated by the fact that there was still significantly more damage than in cells exposed to the respective extracts but not hydrogen peroxide. A possible explanation for this is that the Comet assay is performed in PBS, giving the extracts the opportunity to diffuse out of the cells, thus reducing intracellular protective effects. One interesting observation is that the Maori potato extracts, which were consistently the least protective antioxidant in the chemical assays, appear to be the most protective against ROS induced DNA damage at 0 ºC (table 4). Perhaps a compound exists in the Maori potato which can not scavenge free radicals directly, but triggers the production of other compound within the cells which can. This tells us that the Comet assay is perhaps more reliable than the chemical assays because it is more biologically relevant, using living cells. Therefore, in addition to primary antioxidant activity such as ROS scavenging, the Comet assay is able to detect secondary or indirect antioxidant actions.

Also interesting is the observation that the second best antioxidant in the untreated group, red cabbage extract, now appears to be pro-oxidant. This indicates that, while red cabbage extract does not induce DNA damage directly, it contains other compounds which can enhance the deleterious effects of exogenous ROS in a non-enzymatic manner. Metal ions, for example, are able to synergistically increase the effects of ROS [31].

Whereas performing the hydrogen peroxide challenge at 0 ºC showed direct scavenging of exogenous ROS by the extracts, performing the hydrogen peroxide challenge at 37 ºC also allowed enzymatic effects to be taken into account. Surprisingly the average tail extent moment seen in the 37 ºC hydrogen peroxide challenged controls was reduced back to around the same value as the non-hydrogen peroxide challenged controls. This may suggest that protective antioxidant enzymes such as catalase and glutathione peroxidase, which convert hydrogen peroxide into water and oxygen, as well as DNA repair enzymes, have sufficient protective capabilities to reduce ROS induced damage to background damage[32]. To some degree this was expected in that the treatment at 0 °C was aimed at inactivating these enzymes, to be able to distinguish the antioxidant capacities of the extracts alone, without any contribution from antioxidant or repair enzymes, which is why increased damage was observed in the control at 0 °C. However, the treatment at 37 °C is perhaps more biologically representative of what is really happening in the human body. It was clear from the results that, just because an extract could protect from endogenous free radical damage, it does not mean that the same extract will protect against exogenous ROS.

Previous structure-activity relationships have suggested that antioxidant capacity varies considerably according to the pattern of substitution of the anthocyanin molecule, the presence of acyl groups, and the nature and positions of glycosyl groups [33]. However, new analytical methods make it clear that many of the earlier studies were not identifying all possible anthocyanins. For example, Arapitsas and Turner [34] analysed and tentatively identified anthocyanin species in red cabbage using HPLC/DAD-ESI/Qtrap MS. They used a pressurized liquid technique for extraction, used photodiode array detection to determine the UV/Vis spectral characteristic of the pigments. Electrospray ionization-linear ion trap mass spectrometry allowed the specific determination of the fragmentation patterns of the anthocyanins. They identified twenty four distinct anthocyanins (nine of them newly identified), all having cyanidin as aglycon, but presenting as mono- and/or di-glycoside, and acylated, or not, with aromatic and aliphatic acids.

In our studies, the TRAP assay revealed that AVIs and equimolar free anthocyanins have very similar antioxidant capacities in the short term, with the antioxidant potential of the AVIs only slightly less than that of the free anthocyanins. However, observation of the plate used in the TRAP assay after 24 hours showed that the AVIs, but not the free anthocyanins, had continued to scavenge the ABTS radical. These results show that although AVIs are slower in scavenging free radicals, they have greater total antioxidant activity based on an equimolar anthocyanin concentration. This may be due to the highly organised structure of the AVIs allowing free radicals to be delocalised across the many aromatic rings of the packed anthocyanins.

Although we have not done in vivo studies here, other researchers have reported that consumption of anthocyanins or anthocyanin-rich diets leads to increased serum antioxidant potential in both experimental animals and human subjects [35,36]. Additionally, Ramirez-Tortosa [37] reported that an anthocyanin-rich extract decreased hepatic lipid peroxidation in oxidatively-stressed rats. The present results with AVIs suggest that these structures may be of particular interest in such a model. The association of anthocyanins with plant cell walls, as in the kumara skin [38], may also change in vivo properties and add another dimension to the complexity.

## 3. Experimental Section

### 3.1. Materials

Urenika (purple skin/purple flesh) cultivars were grown in an Auckland University glasshouse and were the kind gift of Dr Kevin Gould, School of Biological Sciences, University of Auckland, Auckland. All other plant materials were field grown. 99N1/222 sweetpotato was the kind gift of Dr Steve Lewthwaite, New Zealand Institute for Crop and Food Research Limited, Mt. Albert, Auckland. Pansies were purchased from Palmers Garden Centre, Glen Eden, Auckland, Blueberries from First Choice Limited, Wiri, Manukau, while red cabbage and red lettuce were both from Foodtown, Greenlane, Auckland. Lisianthus was purchased from In Vogue Blooms, Auckland, New Zealand. The lisianthus used was a deep purple-flowered variety Wakamurasaki, and materials extracted from the even deeper purple throat. During the experiments, fully mature and healthy material was harvested and then thoroughly washed at least three times with 1 liter of tap water containing three drops of Tween-20 prior to a final wash with MilliQ water.

### 3.2. Extract preparation

Approximately 10 mL of extract per sample was prepared in the required solvent (7% acetic acid in methanol for the methanolic extract or Milli-Q water for the aqueous extract) at a concentration of 10% fresh weight per volume. The homogenizer (Ultra-Turrax, Janke & Kunkel Gmbh & Co., Staufen, Germany) tip was washed with the extract solution before use and the plant material was homogenized in a 50 mL conical tube with 5 mL of the extract solution until completely macerated. The homogenized solution was filtered using a Büchi B-169 vacuum system (Büchi Laboratoriums-Technik AG, Switzerland) through a 60 mL F glass filter (Kontes) into a 250 mL vacuum flask (Kimax, USA), both pre-washed in the extract solution. The remaining required volume of extract solution was used to wash both the 50 ml conical tube and the glass filter, before the wash solution was filtered through and combined with the initial filtrate. Extracts were freeze dried and stored at −20 ºC. The methanolic extract is designated anthocyanin-rich extract, or ARE. Anthocyanin levels in the extracts were estimated as A_λmax_ values in the 500–550 nm waveband as measured using a Hitachi dual beam spectrophotometer. The phenolic composition of some of the extracts was determined after 2D-paper chromatography (2D-PC) as described by [19].

### 3.3. DPPH Assay

The methanolic extract was used for these experiments, serially diluted into the extract solution in 96-well plates in 50 μL volumes. One hundred and fifty μL of 100 μM DPPH (1,1-diphenyl-picrylhydrazyl) in methanol was added to all the wells and the plates were left to stand in the dark for 30 minutes. Absorbance values were read at 515 nm using a Spectra MAX Plus plate reader (Molecular Devices, Sunnyvale, CA, USA) and the Softmax Pro 2.4 software. Duplicate absorbencies were averaged and plotted against extract volume. IC_50_ values were calculated as the volume of extract corresponding to the absorbance midway between the highest absorbance on the plate (extract solution and DPPH only) and the lowest absorbance value on the plate (the highest concentration of the ascorbic acid standard).

### 3.4. Total Reactive Antioxidant Potential (TRAP) Assay

The buffer reaction mixture was freshly made for each experiment with 5 mL 750 μM ABTS (2,2′-azinobis (3-ethylbenzothiazoline-6-sulfonate), 10 mL 10 mM AAPH (2,2′-azobis (2-amidinopropane) dihydrochloride) stock solutions and 35 mL 50 mM acetate buffer, pH 4.3. The 50 mM acetate buffer, pH 4.3 was made up of 100 mM acetic acid and 100 mM sodium acetate. The reaction mixture, which is light sensitive, was incubated at 45°C for 60 minutes, then returned to room temperature. Serial dilutions of each aqueous extract were made as for the DPPH assay. Two hundred μL of the reaction mixture was added to all the wells, plates were left to stand in the dark for 15 minutes, and absorbencies were read at 734 nm at 25°C using a Spectra MAX Plus plate reader (Molecular Devices, Sunnyvale, CA, USA) and the Softmax Pro 2.4 software. IC_50_ values were calculated as above.

### 3.5. Oxygen Radical Absorbance Capacity (ORAC) Assay

This was performed as previously described [20]. The methanolic extracts and the antioxidant standard Trolox were dissolved in phosphate buffer (50 mM, pH 7.0) and added to a 96-well plate, followed by the addition of 100 μL of freshly made 3.4 mg/L R-PE (R-phycoerythrin). Plates were incubated at 37°C for 15 min before the addition of AAPH peroxyl radical initiator, to a final concentration of 12 mM. Fluorescence (Ex = 485 nm, Em = 527 nm; F-2000 Fluorescence Spectrophotometer, Hitachi, Ltd., Tokyo, Japan) was recorded every 5 minutes out to 70 minutes. Calculations of ORAC values were made using the formula:

$$\text{S}=(0.5+{\text{f}}_{5}/{\text{f}}_{0}+{\text{f}}_{10}/{\text{f}}_{0}+{\text{f}}_{15}/{\text{f}}_{0}+\ldots +{\text{f}}_{65}/{\text{f}}_{0}+{\text{f}}_{70}/{\text{f}}_{0})\;\;\text{x}\;\;5$$

where f_0_ = fluorescence at 0 minutes

fi= fluorescence at *i* minutes

ORAC values were then calculated from these S values:

$$\text{ORAC}\;\text{Value}\;(\mu \text{M})=20\;k\;({\text{S}}_{\text{sample}}-{\text{S}}_{\text{blank}})/({\text{S}}_{\text{trolox}}-{\text{S}}_{\text{blank}})$$

where *k* = dilution factor (1000)

S=the area under the fluorescence decay curve of the sample

ORAC values were expressed as umol Trolox/g sample.

### 3.6. Single cell gel electrophoresis (Comet) Assay

Human colon cancer (HT-29) cells were maintained in Dulbecco’s Modified Eagle’s Medium (DMEM), supplemented with 10% fetal bovine serum, penicillin (100 unit/ml), and streptomycin (100 mg/ml). Cells were incubated at 37°C in a 5% CO2 incubator, and subcultured before reaching 90% confluency at 2–3 day intervals. Preliminary growth inhibition assays established the concentration of ARE to reduce cell growth by 10% over a three day incubation period (IC_90_), and this concentration was used for subsequent experiments. Cells were incubated at 37 °C for three days, in the presence of the IC_90_ of the appropriate ARE. They were PBS washed, the supernatant discarded and the pellet re-suspended in 2 mL supplemented media and kept on ice while cells were being counted. Cell numbers were determined using a Coulter Cell Counter (Coulter Electronics, Ltd., Luton, Beds, England). Cells were centrifuged (Sorvall RT7 Plus) at 800 rpm for 10 minutes and the supplemented media supernatant discarded before the cells were resuspended in the appropriate volume of PBS to give 1 × 10^6^ cells/mL.

For each of the extract or control treatments, 20 μL of cells were added to three microcentrifuge tubes. The first tube received no treatment and was kept at room temperature, while the second tube received 20 μM hydrogen peroxide diluted in PBS at 0 °C (on ice) while the third tube received 20 μM hydrogen peroxide at 37 °C (in a water bath). After 15 minutes incubation, the tubes were centrifuged at 1,000 rpm for 3 minutes and the supernatants were discarded.

Slides were prepared treated and electrophoresed as described by Singh [21]. DNA damage was visualized using a fluorescence microscope (Zeiss Standard 20, West Germany) using a 25x objective. The Komet 5 software (Kinetic Imaging, Ltd., USA) was used to score 50 cells per slide. Only typical cells per slide were scored, apoptotic cells without visible nuclei were not included. Cells whose head sizes the software was not able to recognize due to their irregular shape, dividing cells or cells that were too close together that their tails interfered with each other, as well as those with tails longer than the window of the software, were also ignored. Comets were visualized and captured using a CCD Camera Model KP-M1E/K-S10 (Hitachi Kokusai Electric, Inc., Japan).

### 3.7. Statistical Analysis

Kolmogorov-Smirnov normality analysis showed that, of the three chemical assays, only the ORAC assay produced normally distributed data (p>0.02). This lack of normality in the DPPH (p=0.003) and TRAP (p<0.001) assay data sets make regression analysis inappropriate to determine whether correlations exist between the various assays. Therefore, the nonparametric method of Spearman rank order correlation was used to determine relationships between data from the chemical assays.

The averages, standard deviations and standard errors of the 100 comets scored for each extract and treatment applied were calculated using Microsoft Excel 2000 (Microsoft Corporation, USA). This information was plotted on bar graphs using the SigmaPlot 2000 software (SPSS, Inc., Chicago, Illinois, USA) with the standard errors being represented by the error bars. Frequency distributions for each extract and treatment applied were also plotted. P-values were calculated with the t-test function from SigmaPlot 2000 (SPSS, Inc., Chicago, Illinois, USA) to compare between the extracts plus the control for each separate treatment. The t-test function was also used to determine the p-values comparing between the 0 ºC and 37 ºC treatment groups. P-values less than 0.05 were taken to indicate a statistically significant difference. Kolmogorov-Smirnov normality tests and Spearman rank order correlations of the three chemical assays and the three Comet treatment groups were performed using SigmaStat v2.03 (SPSS, Inc., Chicago, Illinois, USA).

## 4. Conclusions

Anthocyanins are very complex, and the behaviour of even apparently closely related structures may be different. The primary result of the present studies is that what one assay suggests as their antioxidant potential may be quite different to the results suggested by another assay. Furthermore, the way in which the anthocyanin presents in the cell may be especially important. At least in lisianthus petals, the AVIs in the central vacuoles are derived from the aggregation of anthocyanins into insoluble structures that are similar to membranous networks in appearance. This structural aggregation may lead to significantly different antioxidant properties from those of the isolated molecules. In addition, anthocyanins are typically present in complex mixtures and may interact with one another and with other polyphenols in establishing their biological properties.

## Figures and Tables

**Figure 5.**
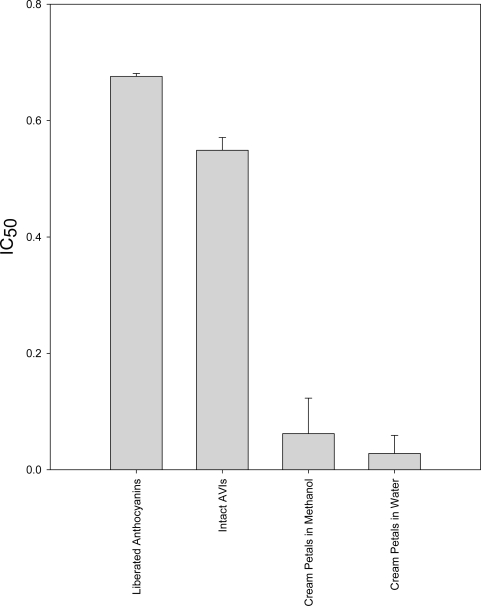
Bar graph showing IC_50_ values for free anthocyanins and anthocyanin released from AVIs, using the TRAP assay. The IC50 values represent the volume of extract required to reduce the absorbance of the TRAP reaction mixture by half, and were calculated from the average of duplicate absorbances.

**Table 1.** Botanical name and major anthocyanidins in some common plants.

**Common name**	**Botanical name**	**Anthocyanidin[s]**	**References**

Red lettuce	*Lactuca sativa L. var. Lollo Rosso*	Cyanidin	[12]
Red cabbage	*Brassica oleracea L. var. capitata f. rubra*	Cyanidin	[12]
Blueberry	*Vaccinium corymbosum L.*	Delphinidin, cyanidin, petunidin, peonidin, and malvidin	[11,13]
Maori potato (flesh)	*Solanum tuberosum L. var Ureniki*	Petunidin, malvidin.	[16,17]
Kumara var. “Rascal” (flesh)	*Ipomoea batatas L. var* 99N1/222	Cyanidin, peonidin.	[14,15]
Kumara var. “Rascal” (skin)	*Ipomoea batatas L. var* 99N1/222	Cyanidin, peonidin.	[14,15]
Deep purple pansy	*Viola x wittrockiana*	Delphinidin.	[10]
Deep purple lisianthus	*Eustoma grandiflorum*	Delphinidin, Cyanidin	[10]

**Table 2.** P-values determined from unpaired t-tests for tail extent moment in the untreated group. Shaded boxes represent significant differences (p ≤ 0.05).

	Control	99N1/222 (skin)	99N1/222 (flesh)	Maori Potato	Pansies	Lisianthus	Blueberries	Red Cabbage	Red Lettuce
Control									
99N1/222 (skin)	0.000								
99N1/222 (flesh)	0.000	0.000							
Maori Potato	0.014	0.000	0.023						
Pansies	0.000	0.000	0.552	0.079					
Lisianthus	0.001	0.000	0.304	0.266	0.603				
Blueberries	0.000	0.004	0.848	0.062	0.755	0.457			
Red Cabbage	0.000	0.002	0.690	0.010	0.331	0.181	0.604		
Red Lettuce	0.098	0.000	0.006	0.400	0.020	0.076	0.016	0.003	

**Table 3.** P-values determined from unpaired t-tests for tail extent moment in the 0 ºC H_2_O_2_ treatment group. Shaded boxes represent significant differences (p ≤ 0.05).

	Control	99N1/222 (skin)	99N1/222 (flesh)	Maori Potato	Pansies	Lisianthus	Blueberries	Red Cabbage	Red Lettuce
Control									
99N1/222 (skin)	0.018								
99N1/222 (flesh)	0.008	0.893							
Maori Potato	0.006	0.724	0.776						
Pansies	0.025	0.704	0.527	0.387					
Lisianthus	0.373	0.037	0.009	0.006	0.053				
Blueberries	0.376	0.000	0.000	0.000	0.001	0.044			
Red Cabbage	0.002	0.000	0.000	0.000	0.000	0.000	0.017		
Red Lettuce	0.759	0.007	0.003	0.002	0.009	0.210	0.572	0.005	

**Table 4.** P-values determined from unpaired t-tests for tail extent moment in the 37 ºC H_2_O_2_ treatment group. Shaded boxes represent significant differences (p ≤ 0.05).

	Control	99N1/222 (skin)	99N1/222 (flesh)	Maori Potato	Pansies	Lisianthus	Blueberries	Red Cabbage	Red Lettuce
Control									
99N1/222 (skin)	0.159								
99N1/222 (flesh)	0.029	0.473							
Maori Potato	0.015	0.288	0.705						
Pansies	0.039	0.642	0.762	0.492					
Lisianthus	0.012	0.262	0.601	0.881	0.407				
Blueberries	0.000	0.000	0.000	0.000	0.000	0.000			
Red Cabbage	0.000	0.001	0.001	0.002	0.001	0.003	0.104		
Red Lettuce	0.002	0.166	0.543	0.863	0.326	0.998	0.000	0.002	

**Table 5.** P-values determined from unpaired t-tests for tail extent moment comparing the 0 ºC and 37 ºC H_2_O_2_ treatment groups. Shaded boxes represent significant differences (p ≤0.05).

Extract	P-Value
Control	0.000
99N1/222 (skin)	0.485
99N1/222 (flesh)	0.932
Maori Potato	0.475
Pansies	0.402
Lisianthus	0.092
Blueberries	0.003
Red Cabbage	0.508
Red Lettuce	0.010
